# Identification of Key Genes Associated With the Process of Hepatitis B Inflammation and Cancer Transformation by Integrated Bioinformatics Analysis

**DOI:** 10.3389/fgene.2021.654517

**Published:** 2021-09-01

**Authors:** Jingyuan Zhang, Xinkui Liu, Wei Zhou, Shan Lu, Chao Wu, Zhishan Wu, Runping Liu, Xiaojiaoyang Li, Jiarui Wu, Yingying Liu, Siyu Guo, Shanshan Jia, Xiaomeng Zhang, Miaomiao Wang

**Affiliations:** ^1^School of Chinese Materia Medica, Beijing University of Chinese Medicine, Beijing, China; ^2^School of Life Sciences, Beijing University of Chinese Medicine, Beijing, China

**Keywords:** hepatitis B, hepatocellular carcinoma, inflammation and cancer transformation, bioinformatics, differentially expressed genes, survival rate, biomarkers

## Abstract

**Background:**

Hepatocellular carcinoma (HCC) has become the main cause of cancer death worldwide. More than half of hepatocellular carcinoma developed from hepatitis B virus infection (HBV). The purpose of this study is to find the key genes in the transformation process of liver inflammation and cancer and to inhibit the development of chronic inflammation and the transformation from disease to cancer.

**Methods:**

Two groups of GEO data (including normal/HBV and HBV/HBV-HCC) were selected for differential expression analysis. The differential expression genes of HBV-HCC in TCGA were verified to coincide with the above genes to obtain overlapping genes. Then, functional enrichment analysis, modular analysis, and survival analysis were carried out on the key genes.

**Results:**

We identified nine central genes (CDK1, MAD2L1, CCNA2, PTTG1, NEK2) that may be closely related to the transformation of hepatitis B. The survival and prognosis gene markers composed of PTTG1, MAD2L1, RRM2, TPX2, CDK1, NEK2, DEPDC1, and ZWINT were constructed, which performed well in predicting the overall survival rate.

**Conclusion:**

The findings of this study have certain guiding significance for further research on the transformation of hepatitis B inflammatory cancer, inhibition of chronic inflammation, and molecular targeted therapy of cancer.

## Introduction

Epidemiological studies have shown that chronic low-level inflammation can significantly increase the risk of cancer. On the one hand, during chronic inflammation caused by viral infections, a long-term abnormal expression of related proteins may induce physiological diseases and form a potential carcinogenic microenvironment. On the other hand, the occurrence and development of tumors also affect the inflammatory response process (Colotta et al., 2009; Huang et al., 2019). The global burden of hepatitis B virus (HBV) is enormous, with 257 million people chronically infected, causing more than 880,000 deaths worldwide each year (Iannacone and Guidotti, 2021). HBV has all the characteristics of ancient human pathogens, has chronic infections, including a prolonged asymptomatic period, and then gradually develops into clinical diseases. Persistent antiviral inflammation during chronic infection, immune clearance of virally infected cells, and hepatocyte regeneration all increase the risk of viral infectious liver disease developing into liver cancer (Xu et al., 2017; Revill et al., 2020). At present, vaccines and nucleoside or nucleotide drugs have been developed, with high coverage and efficacy. However, related studies have shown that vaccination and antiviral therapy can reduce infections but not completely eliminate risks, and reduce the rate of new infections and the development of liver disease (Chan et al., 2016; Fanning et al., 2019; Musa et al., 2019). Overall, up to 40% of men and women infected with HBV during the perinatal period will die from cirrhosis or hepatocellular carcinoma (Trépo et al., 2014; Schweitzer et al., 2015). There are many studies on the basis of clinical epidemiological studies on the mechanism of the relationship between hepatitis B and hepatitis B-related hepatocellular carcinoma, and significant progress has been made (Huang et al., 2019; Sun et al., 2019; Xie et al., 2020). However, few molecular targeted studies can comprehensively summarize the diagnosis, treatment, and prognosis of patients with progressive hepatitis B.

**FIGURE 1:**
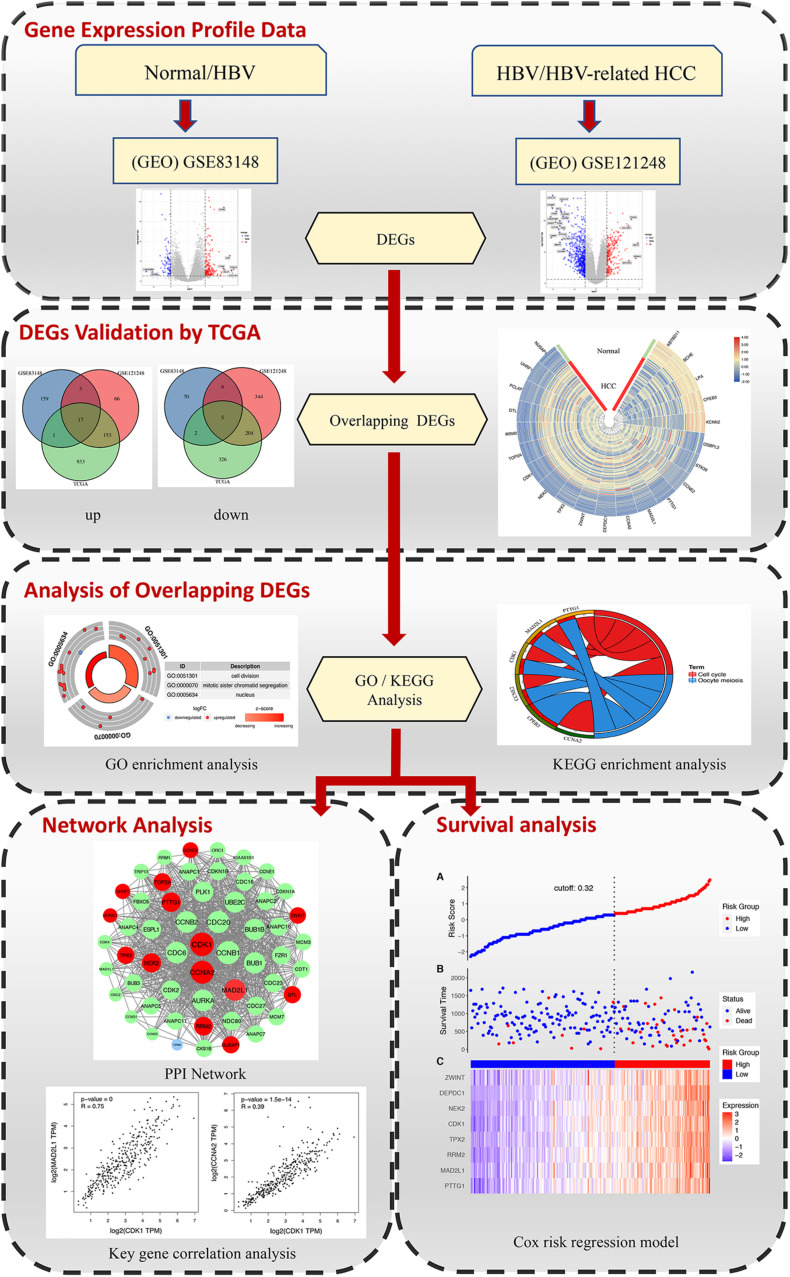
The workflow for identifying key genes associated with HBV in inflammation and cancer transformation.

The rise of high-throughput gene chips and transcriptome sequencing and other transcriptome research methods has completely changed the previous systematic analysis methods for disease research (Kulasingam and Diamandis, 2008; Mair et al., 2019; Bustoros et al., 2020). High-throughput microarrays and RNA sequencing can detect changes in disease gene expression and transcriptome levels. These methods help to find reliable biological markers, classify diseases, and reveal the molecular mechanisms of disease development (Chen S. et al., 2020; Jin et al., 2020; Li X. et al., 2020). The purpose of this study is to find the key genes in the process of liver inflammation and cancer transformation and to provide reference for further study of the transformation of hepatitis B inflammatory cancer, inhibition of chronic inflammation, and molecular targeted therapy of cancer. In this study, we conducted a comprehensive analysis, selecting microarray data of normal tissues and HBV samples and microarray data of chronic hepatitis B-induced HCC and adjacent normal tissues, and separately analyzing the differentially expressed genes (DEGs) of the two groups of gene chips. Combining the TCGA DEG data of human hepatitis B-related hepatocellular carcinoma and normal liver tissue with the abovementioned chip data to obtain the key DEGs that directly affect the diagnosis and treatment of hepatitis B and later. Afterward, further functional enrichment analysis was conducted to analyze the main biological functions regulated by DEGs. Finally, through the use of protein–protein interaction (PPI) networks and survival analysis, key genes affecting the diagnosis, treatment, and prognosis of patients with progressive hepatitis B are identified. The detailed workflow of the study is shown in Figure 1.

## Materials and Methods

### Gene Expression Profile Data

Gene expression profiles were extracted from the GSE83148 and GSE121248 data set, which was downloaded from the publicly available Gene Expression Omnibus database (GEO)^1^ (Clough and Barrett, 2016). GSE83148 (Zhou et al., 2017; Li et al., 2018; Chen Z. et al., 2019) and GSE121248 (Wang et al., 2007) are both based on the GPL570 ([HG-U133_Plus_2] Affymetrix Human Genome U133 Plus 2.0 Array). The title of the GSE83148 data set is “Expression data of HBV infected liver tissue.” All hepatitis samples were HBV infected, which was validated by positive HBsAg or serum HBV-DNA. The samples with HCV infection or metabolic liver injury (e.g., fatty liver, chronic alcoholic hepatitis) were excluded. GSE83148 contains six human normal liver tissue samples and 122 HBV-infected hepatitis samples. The title of the GSE121248 data set is “Gene expression profiling of chronic hepatitis B induced HCC and adjacent-normal tissues.” Tissues from chronic hepatitis B-induced HCC and their adjacent normal tissues were isolated, and total RNA was extracted for Affymetrix gene microarray analysis. GSE121248 contains 37 chronic hepatitis B-induced HCC adjacent normal tissues and 70 human chronic hepatitis B-induced HCC liver tissues.

### Screening of DEGs and Functional Enrichment Analysis

The limma software package in the R 3.6.3 software^2^ was used to normalize the matrix data of each GEO data set and the logarithm conversion based on 2. The DEG between the two groups of controls was screened through the limma software package. Corrected *p* < 0.05 and | log FC| > 1 were used as the cutoff criteria (Ritchie et al., 2015; Zhou et al., 2020). After that, functional enrichment analyses were performed on the obtained differential genes, including Gene Ontology (GO) and Kyoto Encyclopedia of Genes and Genomes (KEGG) (Rhee et al., 2008; Kanehisa et al., 2017). The DEGs of GO and KEGG pathway analysis were performed using Bioconductor clusterProfiler, org.Hs.eg.db, and DOSE, which are three R packages used for the enrichment analysis of gene clusters (Yu et al., 2012; Zou et al., 2019). *p* < 0.05 and *q* < 0.05 were defined as the cutoff criteria. In the GO analysis, *p* < 0.01 and *q* < 0.05 were defined as the cutoff criteria. Furthermore, in the KEGG analysis, *p* < 0.05 and *q* < 0.05 were defined as the cutoff criteria.

### DEG Validation by TCGA

Using the RNA sequencing data in the TCGA HBV-related HCC data set, the results of the comprehensive analysis of the differential genes in the two GEO data sets were verified (Liu et al., 2018). The TCGA-Liver Hepatocellular Carcinoma (HCC) cohort with publicly available data^3^ was used for this study. From this cohort, 78 HCC cases with gene expression data set, epigenetic data, and copy number alteration data were selected. It contained 60 cases of HBV-related HCC, and 18 cases were HBV-related adjacent tissues. The above data were analyzed by the edgeR software package in the Sanger box.^4^ Genes with | log FC| > 1 and FDR < 0.05 are considered significant (FDR: false discovery rate). The common up-and-down overlapping genes between TCGA and the two GEO data sets were integrated for the next study. These genes are considered to be overlapping genes related to the occurrence and development of hepatitis B-related inflammation and cancer transformation. The obtained overlapping DEGs were visualized by TBtools for heat map analysis (Chen S. et al., 2020).

### GO and KEGG Pathway Enrichment Analysis of Key Genes

To elucidate potential biological processes, molecular functions, cellular components, and signaling pathways associated with the overlapping DEGs, we performed GO enrichment analysis and KEGG enrichment analysis utilizing the Database for Annotation, Visualization and Integrated Discovery^5^ (DAVID 6.8) (Dennis et al., 2003; Huang da et al., 2009). FDR < 0.05 was defined as the cutoff criterion. The results of the GO functional enrichment analysis were visualized *via* GOplot software package in the R 3.6.3 software (Walter et al., 2015). The results of KEGG functional enrichment analysis are drawn by Sanger box.^6^

### PPI Network and Module Analysis

The String 11.0 database^7^ is a database that searches for interactions between known proteins and predicted proteins (Szklarczyk et al., 2017). The database is used to study PPI networks, which helps to mine core regulatory genes. In this study, we selected protein interaction results with confidence greater than 0.7 for the next analysis. Data of protein interaction were imported into Cytoscape 3.7.1^8^ for visual analysis (Franz et al., 2016). In addition, in order to detect the hub cluster module in the PPI network, we used the Molecular Complex Detection (MCODE) application with default parameters in Cytoscape 3.7.1 for module analysis (Zhang et al., 2020).

### Expression Level Analysis and Correlation Analysis of the Key Genes

The violin diagram tool in Sanger Box^9^ was used to show the difference in the expression of key genes in HBV-related HCC tissues and normal tissues. Gene Expression Profiling Interactive Analysis^10^ (GEPIA) is the dynamic analysis of gene expression profiling data. It is a newly developed public database for cancer and normal gene expression profiling. GEPIA analyzed the RNA sequencing expression data of 9,736 tumors and 8,587 normal samples from TCGA and GTEx projects (Tang et al., 2017). Perform pairwise gene correlation analysis on any given TCGA and/or GTEx expression dataset and check the relative ratio between the two genes.

### Survival Analysis

Clinical information for patients with hepatocellular carcinoma can also be downloaded from TCGA (see text footnote 3). After screening HBV-related HCC, after deleting patients without overall survival (OS) data and overlapping DEG gene expression profiles, 60 patients with HBV-related HCC were used for survival analysis. Univariate Cox proportional hazards regression analysis was used to identify candidate genes that were highly correlated with survival. Cox proportional hazards regression analysis screened prognostic gene signatures from DEGs, *p* < 0.05. A Cox proportional hazards regression model was constructed with key prognostic genes as dependent variables, with the purpose of evaluating the relative contribution of key prognostic genes to patient survival prediction. We have constructed a prediction formula for gene characteristics. The following formula of the model is as follows: risk score = gene 1 × β1 gene 1 expression + gene 2 × β2 gene 2 expression + … gene n × βn expression gene. The formula is a linear combination in which the gene expression value of each gene and the regression coefficient (β) were obtained from the multiple Cox proportional hazards regression model (George et al., 2014; Zhou et al., 2016; Huang et al., 2017, 2018; Liu et al., 2019). The survminer package and ggrisk package in the R language were used to draw a riskplot and K-M survival curves (Cox, 1972; Zhou et al., 2016; Li X. et al., 2020). The LIRI data were downloaded in the ICGC database,^11^ and 260 primary solid tumor tissue samples were extracted. Samples with complete expression profile data and clinical information were selected, and RNA-seq data and clinical information of 231 tumor samples were obtained. These samples were mainly from Japanese people with hepatocellular carcinoma, and the FPKM values from genes were used. The data in ICGC were taken as the test set. Nine prognostic genes in the current TCGA were selected as the test set, the training set and the test set were modeled, and the model was verified (He et al., 2020; Liang et al., 2020). In order to analyze the accuracy of survival prediction performance through a risk scoring model, a time-dependent receiver operating characteristic (ROC) curve was constructed. The ability of prognostic gene signatures to predict clinical outcome depends on the area of the AUC curve. When AUC > 0.5, the closer AUC is to 1, the better the prognosis (Heagerty and Zheng, 2005).

## Results

### Identification of DEGs

**FIGURE 2:**
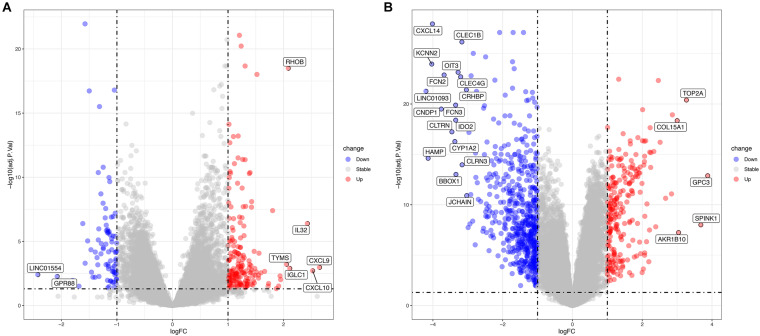
**(A)** Volcano map of differential genes in the GSE83148 data set. **(B)** Volcano map of differential genes in the GSE121248 data set. Blue indicates down-regulated genes, red indicates up-regulated genes.

The GSE83148 data set includes six human normal liver tissue samples and 122 HBV-infected hepatitis samples. Supplementary Table 1 and Figure 2A show the results of the differential analysis of the GSE83148 data set, including 263 DEGs, 83 down-regulated genes, and 180 up-regulated genes. GSE121248 contains 37 chronic hepatitis B-induced HCC adjacent normal tissues and 70 human chronic hepatitis B-induced HCC liver tissues. Supplementary Table 2 and Figure 2B show the results of the differential analysis of the GSE121248 data set, including 798 DEGs, 559 down-regulated genes, and 239 up-regulated genes.

### Enrichment Analysis of Two Groups of DEGs

**FIGURE 3:**
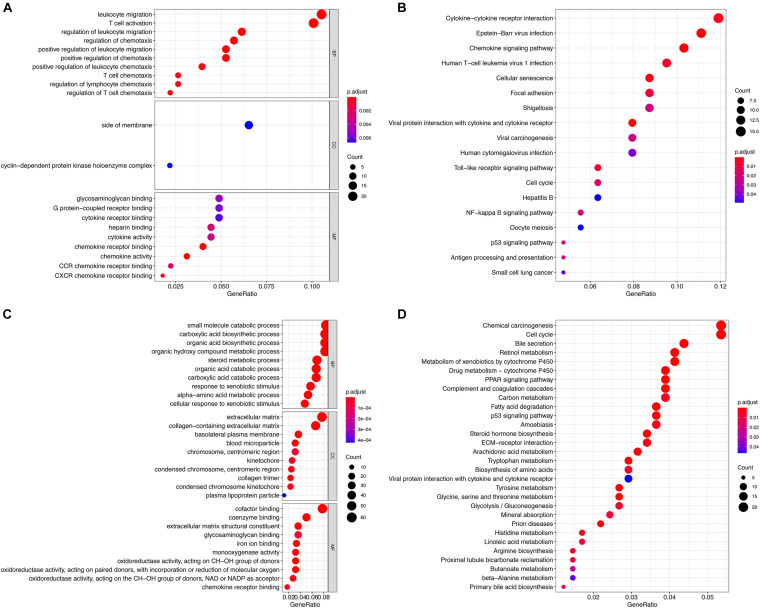
Functional enrichment analysis of the two groups of DEGs. **(A)** GO analysis of DEGs in the GSE83148 data set. **(B)** KEGG analysis of DEGs in the GSE83148 data set. **(C)** GO analysis of DEGs in the GSE121248 data set. **(D)** KEGG analysis of DEGs in the GSE121248 data set.

The two groups of DEGs in GSE83148 and GSE121248 were analyzed by GO and KEGG enrichment, respectively (Figures 3A–D). The DEGs in the GSE83148 data set are enriched in different functional entries. In GO analysis, DEGs are mainly enriched in the entry of leukocyte migration in the biological process (BP), mainly in the side of membrane in terms of cell component (CC), and mainly in glycosaminoglycan binding in terms of molecular function (MF). According to KEGG pathway enrichment analysis, the DEGs are mainly enriched in the cytokine–cytokine receptor interaction pathway, cell cycle pathway, hepatitis B pathway, oocyte meiosis pathway, viral carcinogenesis pathway, etc. Then, in the DEG enrichment analysis of GSE121248, DEGs were mainly enriched in the organic acid catabolic process, extracellular matrix, and cofactor binding in BP, CC, and MF. In addition, in the KEGG pathway, DEGs are mainly enriched in chemical carcinogenesis pathway, cell cycle pathway, etc. The results of KEGG pathway enrichment suggested that there were two identical pathways in the two data sets, including cell cycle pathway and P53 signaling pathway.

### Overlapping DEGs

**FIGURE 4:**
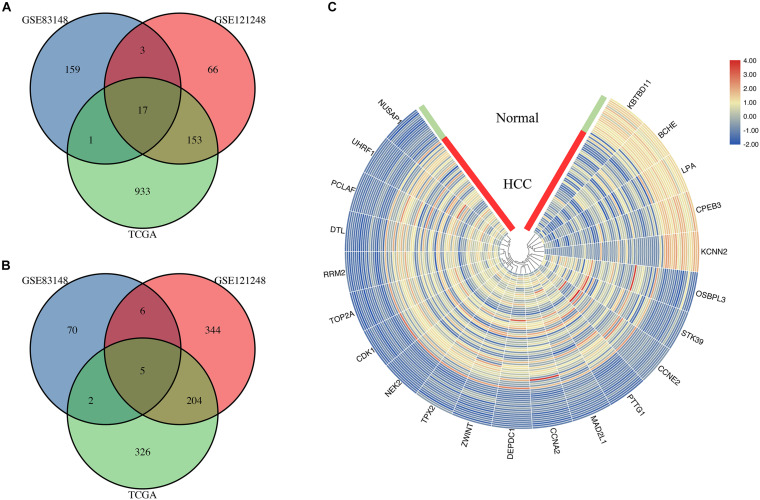
Identification of genes common to chronic HBV infection and hepatocellular carcinoma. **(A)** The Venn diagram of the up-regulated DEGs between the two GEO data sets and the TCGA HBV-HCC data set (drawn by SangerBox, http://sangerbox.com/Signin). **(B)** The Venn diagram of the down-regulated DEGs between the two GEO data sets and the TCGA HBV-HCC data set (drawn by SangerBox, http://sangerbox.com/Signin). **(C)** The heat map of 5 down-regulated DEGs and 17 up-regulated DEGs in the integrated microarray analysis. The green band in the figure represents the normal group samples, and the red band represents HBV-related HCC samples. The gradual color ranging from blue to red represents the changing process from down-regulation to up-regulation.

**FIGURE 5:**
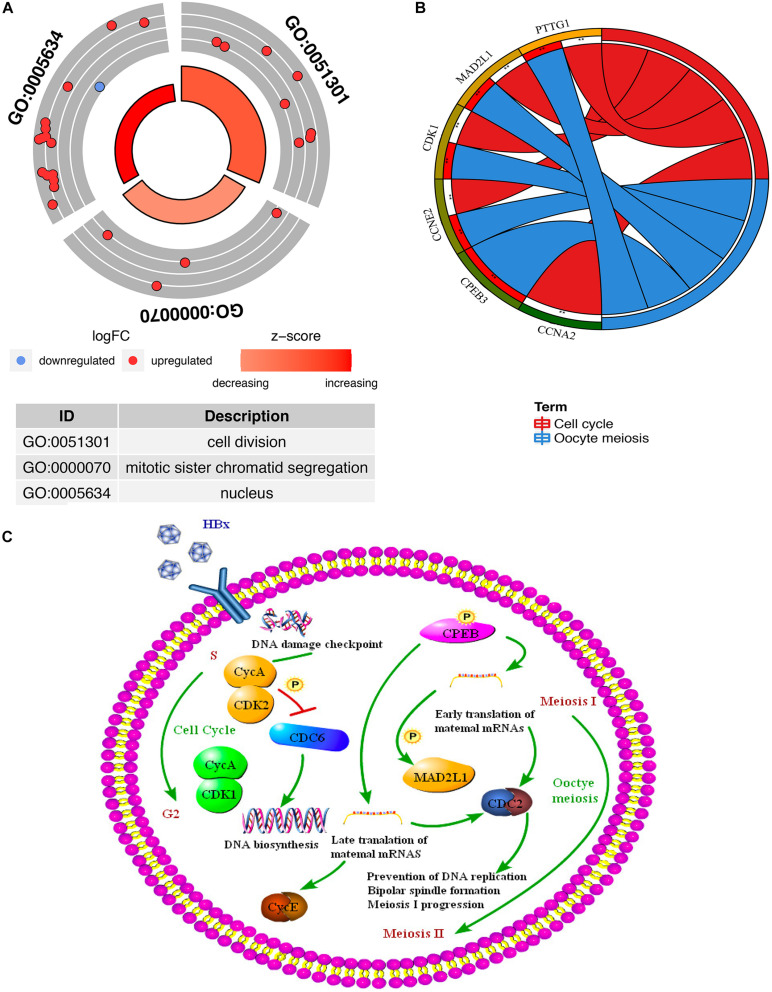
Functional enrichment analysis of the overlapping DEGs (genes common to chronic HBV infection and hepatocellular carcinoma). **(A)** GO enrichment analysis of the overlapping DEGs. Red indicates the up-regulated gene, and blue indicates the down-regulated gene. The thicker the red circle in the middle of the graph, the more significant the difference, and the darker the red, the greater the proportion of up-regulated genes in the entry. **(B)** GO enrichment analysis of the overlapping DEGs. The right side of the outermost circle is the term, and the color corresponding to the gene on the left is the gene expression multiple. The inner circle on the left indicates the significance *p* of the gene corresponding pathway. **(C)** The key targets and key biological processes involved in hepatitis B-related inflammation and cancer transformation.

In the mRNA sequencing data in TCGA, 60 HBV-positive HCC and 18 HBV-related adjacent tissue RNA-seq reading count data were screened. The clinical characteristics of all patients are shown in Supplementary Table 3. The results of the DEG analysis of TCGA are listed in Supplementary Table 4. By sequencing TCGA HBV-related HCC, 1,641 DEGs were obtained, including 1,104 up-regulated genes and 537 down-regulated genes. The DEGs in the above two HBV-related liver disease gene chip data sets and the genes identified as differentially expressed in the TCGA HBV-related HCC sequencing data set are taken to intersect to screen for common overlapping DEGs. The common overlapping DEGs were screened. Figure 4 shows that a total of 22 overlapping DEGs were obtained, including 17 overlapping up-regulated DEGs (Figure 4A) and 5 overlapping down-regulated DEGs (Figure 4B). They may be the DEGs during the progression of HBV infection to HBV-related hepatocellular carcinoma. The visual analysis of 22 overlapping DEGs was performed by TBtools (Figure 4C). The green band in the figure represents the normal group samples, and the red band represents HBV-related HCC samples. The gradual change of color from blue to red represents the process of gene down-regulation to up-regulation.

### Functional Annotation of Overlapping DEGs by GO and KEGG Pathway Analyses

Through the GO and KEGG pathway analysis, 22 overlapping DEGs were functionally annotated to clarify their potential biological functions. GO analysis of overlapping DEGs induced by HBV was enriched in items with significant differences (Figure 5A and Supplementary Table 5). These three entries are cell division, mitotic sister chromatid segregation, and nucleus. To further analyze the pathogenic mechanism of HBV, KEGG pathway analysis was performed on the identified overlapping DEGs (Figure 5B and Supplementary Table 6). The results showed that the overlapping DEGs were mainly enriched on the oocyte meiosis pathway and cell cycle pathway (Figure 5C).

### Key Gene Analysis

#### PPI Network and Module Analysis

**FIGURE 6:**
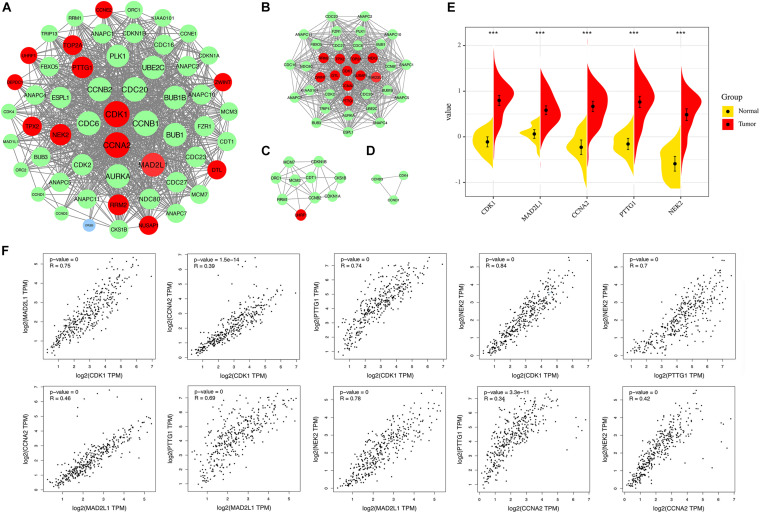
**(A)** The PPI network of overlapping DEGs (genes common to chronic HBV infection and hepatocellular carcinoma). **(B)** Module 1 (MCODE score = 29.722). **(C)** Module 2 (MCODE score = 6.222). **(D)** Module 3 (MCODE score = 3). Blue circles represent down-regulated genes, red circles represent up-regulated genes, and green circles indicate secondary proteins obtained by protein interaction. **(E)** Expression of the five key DEGs in HBV-related HCC and normal tissues (TCGA data set). **(F)** Correlation analysis of five key genes. From left to right, from top to bottom: CDK1-MAD2L1, CDK1-CCNA2, CDK1-PTTG1, CDK1-NEK2, PTTG1-NEK2, MAD2L2-CCNA2, MAD2L1-PTTG1, MAD2L1-NEK2, CCNA2-PTTG1, CCNA2-NEK2.

A PPI network was constructed in the STRING 11.0 database, including 56 nodes and 869 interactions. As shown in Figure 6A, the size of the node is proportional to the degree value. Red nodes indicate up-regulated genes, blue nodes indicate down-regulated genes, and green nodes indicate secondary proteins obtained by protein interaction. The protein interaction results show that the nodes with interactions are mainly up-regulated genes. The top 5 genes with the highest degree are considered key genes, and they are all up-regulated genes. In addition, this study uses MCODE plug-in in Cytoscape to analyze the PPI network module and obtains important cluster modules. A total of three modules were obtained (Figure 6B–D). Module 1 has the highest score of 29.722. In addition, five key genes are concentrated in module 1, which also indicates that it may be the main functional module. The score of module 2 is 6.222, and the score of module 3 is 3. The five key genes include cyclin-dependent kinase 1 (CDK1), mitotic spindle assembly checkpoint protein MAD2A (MAD2L1), Cyclin-A2 (CCNA2), Securin (PTTG1), and serine/threonine-protein kinase Nek2 (NEK2). They are defined as the main hub nodes in the PPI network; the change in gene expression is shown in Figure 6E.

#### Correlation Analysis of Key Gene Expression Levels

We applied GEPIA to capture the correlation of expression levels between key genes. Correlation analysis was conducted on any two genes of CDK1, MAD2L1, CCNA2, PTTG1, and NEK2 and five key genes (Figure 6F). The results showed that the significance between any two genes was *p* < 0.01, indicating that the correlation coefficient was statistically different. The larger the correlation coefficient “R” is, the better the correlation between the two genes is. The four results (CCNA2-CDK1, CCNA2-MAD2L1, PTTG1-CCNA2, CCNA2-NEK2) are relatively weakly correlated (*R* < 0.5), but *p* is still extremely low. The above results indicate that the up-regulation of one of them will affect the high expression of other genes. This may indicate that they are all regulated by the same transcription factors and epigenetic modifications.

### Survival Analysis

#### Cox Regression Analysis

**FIGURE 7:**
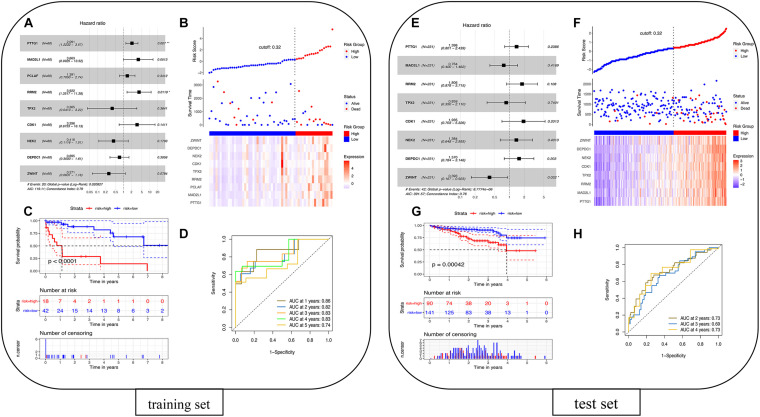
Prognostic analysis of the nine-gene signature model in the TCGA cohort. **(A)** Training set forest diagram. **(B)** Risk score in the TCGA cohort. **(C)** Kaplan–Meier curve of OS for patients in the high-risk group and low-risk group. **(D)** The AUC of the time-dependent ROC curve in the TCGA cohort. Validation of the eight-gene signature in the ICGC cohort. **(E)** Test set forest diagram. **(F)** Risk score in the ICGC cohort. **(G)** Kaplan–Meier curve of OS for patients in high-risk group and low-risk group. **(H)** The AUC of the time-dependent ROC curve in the ICGC cohort.

The univariate Cox proportional hazards regression model was used to analyze 22 overlapping DEGs, and nine genes that were significantly related to survival time were identified (*p* < 0.05). After using the multivariate Cox proportional hazards regression model, a prognostic gene signature consisting of nine genes was developed, including Securin (PTTG1), mitotic spindle assembly checkpoint protein MAD2A (MAD2L1), PCNA-associated factor (PCLAF), ribonucleoside-diphosphate reductase subunit M2 (RRM2), targeting protein for Xklp2 (TPX2), cyclin-dependent kinase 1 (CDK1), serine/threonine-protein kinase Nek2 (NEK2), DEP domain-containing protein 1A (DEPDC1), and ZW10 interactor (ZWINT) (Supplementary Table 7). Figure 7A shows the forest plot of Cox regression. Using the survminer software package to multiply gene expression by the linear combination regression coefficient obtained through multiple Cox regression, the optimal cutoff threshold can be calculated, and more suitable high-risk groups and low-risk groups can be obtained. The risk scores of the patients are ranked, and then the survival status of the patients is displayed through a dot graph, and the expression of nine prognostic genes is displayed through a heat map. Figure 7B shows the patients’ risk scores in order from low to high: red indicates the high-risk group, and blue indicates the low-risk group. Different patients have different survival times: blue indicates survival during follow-up, and red indicates death during follow-up. The heat map of the nine prognostic genes shows that as the risk value increases, the survival time of patients tends to be shortened, the proportion of deaths tends to increase, and the nine prognostic genes tend to be highly expressed. The K-M curve in Figure 7C shows the relationship between patient survival time and survival probability (*p* < 0.0001, statistically significant). The red and blue solid lines represent the changes in survival rates of the high-risk group and the low-risk group, and the dotted lines represent the 95 and 5% confidence intervals. Figure 7D is the ROC curve of the patient in the training set. The results showed that the AUC of 1-, 2-, 3-, 4-, and 5-years OS were 0.86, 0.82, 0.83, 0.83, and 0.74, respectively, so the prognostic gene characteristics showed good performance in survival prediction.

#### Cox Model Verification

The 260 primary solid tissue tumor samples can be screened from the ICGC portal website^12^ (Supplementary Table 8). Samples with complete expression profile data and clinical information were selected, and RNA-seq data and clinical information of 231 tumor samples were obtained. These samples were mainly from Japanese people with hepatocellular carcinoma, and the FPKM values from genes were used. Sixty patients in TCGA were taken as the training set, and 231 patients in ICGC were taken as the test set for modeling. Because the PCLAF gene was not found in the test set, the remaining eight genes were thus used for fitting the model in the test set. The coefficients of the training set were extracted, and the expression of eight genes in the test set was multiplied to verify the model. The survminer package was used to recalculate the optimal cutoff threshold to obtain a more suitable high- and low-risk group. The risk score can be calculated by multiplying the gene expression by the linear combination regression coefficient obtained through multiple Cox regression. Figure 7E shows the forest plot of Cox regression. The survminer software package was used to recalculate the optimal cutoff threshold to obtain more suitable high-risk and low-risk populations. Figure 7F shows that the cutoff is 0.32, and the patients’ risk scores are sorted from low to high: red represents the high-risk group, and blue represents the low-risk group. Different patients have different survival times: blue indicates survival during follow-up, and red indicates death during follow-up. The heat map of the eight prognostic genes shows that as the risk value increases, the survival time of patients tends to be shortened, the proportion of deaths tends to increase, and the eight prognostic genes tend to be highly expressed. The K-M curve in Figure 7G shows the relationship between patient survival time and survival probability (*p* = 0.00042, statistically significant). The red and blue solid lines represent the changes in survival rates of the high-risk group and the low-risk group, and the dotted lines represent the 95 and 5% confidence intervals. Figure 7H shows the ROC curve of patients in the training set. The results showed that the AUC of the 2-, 3-, and 4-year OS were 0.73, 0.69, and 0.73, respectively, so the prognostic gene signatures showed good performance in survival prediction. The results show that the area under the ROC curve in the training set and the test set is more than 0.5, and the model is better.

## Discussion

The risk factors of chronic hepatitis B disease progression can be divided into three categories: host factor element, virus factor, and liver factor (Wong et al., 2013). Host factors include age, male, family history of liver cancer, obesity, genetic susceptibility, smoking, alcoholism, diabetes, and immune status (Wong and Janssen, 2015). The viral factors include HBsAg positive, HBeAg positive, high level of HBV DNA, HBV genotype, and HBV mutant. Especially, high viral load is an independent and effective predictor (Wong and Sung, 2012). Liver factors include progressive fibrosis and cirrhosis, poor liver function, hepatitis activity, and other accompanying liver diseases, such as hepatitis C virus or coinfection of alcoholic and non-alcoholic fatty liver (Wong and Wong, 2013). Inflammatory reaction of the liver caused by virus replication in patients with chronic HBV infection is the main factor of liver disease progression. Chronic hepatitis B-cirrhosis-hepatocellular carcinoma is a common law of disease development and transformation in clinic, and its disease transformation process can also be regarded as a typical “inflammatory cancer transformation” process. Finding the key genes in the process of inflammatory cancer transformation is helpful to inhibit the progression of chronic hepatitis B and the transformation from chronic inflammation to cancer. The analysis of comprehensive bioinformatics mainly focuses on the screening of DEGs, the construction of related protein interaction networks, the screening and survival analysis of key genes, and the analysis of gene association. The above method has been widely used to identify potential biomarkers related to the diagnosis, treatment, and prognosis of HBV and HCC. Xie et al. (2020) studied the genetic characteristics of HBV positive (HBV +) HCC and revealed its potential carcinogenic mechanism by using the methods of differential gene screening, functional enrichment analysis, protein interaction network construction, survival analysis, immunohistochemistry, and statistical analysis. Sun et al. (2019) characterized the genome size of HBV and HCV-infected HCC by comparing the publicly available data of the Cancer Genome Atlas Project (TCGA), comparing their gene expression patterns, methylation profiles, and copy number variation. Huang et al. (2019) identified common gene disorders between HBV and HCC by screening DEGs. In addition, through modular methods such as PPI networks and hypergeometric tests, targeted drugs with regulatory effects on diseases are predicted.

In this study, we obtained two microarray data sets about hepatitis progression and integrated them. GSE83148 identified 263 DEGs between the normal group and HBV, including 180 up-regulated genes and 83 down-regulated genes; GSE121248 identified 798 DEGs of HBV and HBV-related HCC, including 239 up-regulated genes and 559 down-regulated genes. The above two microarray data sets were integrated with TCGA’s RNA sequencing data to identify 22 DEGs, including 17 down-regulated genes and 5 up-regulated genes. They are considered to be differential genes that jointly affect the occurrence and development of hepatitis B inflammatory cancer transformation. GO analysis of overlapping differential genes induced by HBV showed that the differential genes were enriched in cell division, mitotic sister chromatid separation, and nuclear entries. The results of KEGG pathway enrichment analysis showed that overlapping differential genes were mainly enriched in meiosis pathway and cell cycle pathway of oocytes.

In addition, we also identified five key genes in the PPI network and module analysis, namely, CDK1, MAD2L1, CCNA2, PTTG1, and NEK2. Coincidentally, they are all up-regulated genes in HBV disease changes. The CDK family is a Ser/Thr kinase system that corresponds to the cell cycle progression. Various CDKs are alternately activated along the cell cycle phase, phosphorylating the corresponding substrate. Through synergy with cyclin, cell cycle events proceed in an orderly manner. The activity of CDK1 is closely related to the content of CyclinB. CyclinB is generally synthesized in the late G1 phase. Through the S phase and G2 phase, the CyclinB content reached a certain level, entered the nucleus, and bound to CDK1. Then, CDK1 kinase activity began to appear (Yang et al., 2011; Hu et al., 2018). Activated CDK1 can phosphorylate target proteins to produce corresponding physiological effects, such as phosphorylation of nuclear laminin leading to the disintegration of nuclear fibrils, disappearance of nuclear membrane, and phosphorylation of histone H1, leading to the condensation of chromosomes. The final result of these effects is to keep the cell cycle running. Li Y. et al. (2019) also found that CDK1 is an important biomarker in the study of lncRNA-related comprehensive analysis of the ceRNA network to reveal potential biomarkers for the prognosis of HBV-related HCC. They verified the up-regulation of CDK1 in liver cancer in the microarray data set and TCGA database (Li Y. et al., 2019). In addition, some researchers believe that HBx is different from other viral proteins. HBx can continuously activate the cyclin B1-CDK1 kinase. However, the results of some researchers indicate that HBx induces G2/M arrest and apoptosis, which in turn inhibits the growth of HCC cells and vascular endothelial cells *in vitro* and *in vivo*. Another part of the researchers believed that HBx accelerates the appearance of cells entering the S phase from Go/Gland by promoting the rapid and strong activation of CDK kinase activity. HBx may promote viral carcinogenesis through molecular mechanisms (Benn and Schneider, 1995; Cheng et al., 2009; Kim et al., 2015). The contradiction between them may be due to differences of experimental environment and experimental materials, or the different expression levels of HBx. High HBx expression leads to cell cycle arrest and apoptosis, while low HBx expression indicates adverse effects (Bréchot et al., 2000). The results of the study reflect that the persistent chronic expression of HBX may be an important factor in the final progression of HBV to HCC. Mitotic arrest defect protein 2 (MAD2), also known as mitotic spindle assembly checkpoint protein, is encoded by the MAD2L1 gene (Chen Z. et al., 2019). Moreover, it has been reported that MAD2 and CDC20 form mitotic checkpoint complexes to monitor the attachment process of mitochondria spindle and inhibit the activity of late-stage promoting complexes (Luo et al., 2000). It regulates the mitotic process of cells and then affects the malignant progression of a variety of tumors (Fang et al., 1998; Guo et al., 2020). For example, the work of researchers using integrated bioinformatics analysis has shown that MAD2L1 may be a potential therapeutic target for HCC (Yang et al., 2019). The results of another study showed that MiR-200c-5p inhibited the proliferation, migration, and invasion of HCC cells by down-regulating MAD2L1 (Li et al., 2017). This indicates that the expression of MAD2L1 in HCC is significantly higher and is related to poor prognosis. Cyclin A2 (CCNA2) is a member of the cyclin family. Different cyclins can selectively activate specific substrates and cause different cell cycle events (Manni et al., 2001; Loog and Morgan, 2005; Chen et al., 2018). The researchers believe that the overexpression of CCNA2 is related to the carcinogenesis of the liver. There are many exons in CCNA2, and HBV integration occurs in introns. Because cyclin is important in controlling cell division, disrupting the cyclin A gene through viral insertion may help in tumorigenesis (Wang et al., 1990; Bayard et al., 2018). PTTG1 may work by blocking key proteins. Its gene product has *in vitro* transformation activity and *in vivo* tumorigenic activity and is highly expressed in various tumors (Zou et al., 1999). PTTG1 may play a role by blocking the key protein, and its gene product has *in vitro* transformation activity and *in vivo* tumorigenicity and is highly expressed in various tumors. Many studies have also proved that PTTG1 may be an important gene for HBV-related hepatitis to progress into hepatocellular carcinoma (Lin et al., 2019; Shen et al., 2019). The results of Li et al. (2013) suggested that the loss of miR-122 expression will lead to the up-regulation of its target PBF, thereby initiating the nuclear translocation of PTTG1 and promoting the transcriptional activity of PTTG1, thereby enhancing cell growth and invasion. With the development of chronic hepatitis B to cirrhosis and HCC, PTTG1 expression increased. *In vitro* experiments showed that HBx induced significant accumulation of PTTG1 protein without affecting the level of its mRNA. This may provide new insights for the pathogenesis of HBV-related inflammatory cancer transformation (Molina-Jiménez et al., 2010). NEK2 is involved in the control of centrosome separation and bipolar spindle formation in mitotic cells and chromatin condensation in meiotic cells (Hames and Fry, 2002). Researchers such as Xie identified important genes and pathways related to HBV-related HCC through bioinformatics analysis and found that NEK2 is a key gene in the protein interaction network. This is very similar to our results (Xie et al., 2019). Cheng et al. (2018) found in a cohort study that a high expression of NEK2 was an independent risk factor for decreased OS. The results of the study suggest that a high expression of NEK2 is a risk factor for poor survival of liver cancer patients (Cheng et al., 2018; Ren et al., 2018).

The current study identified nine key genes for prognosis of HBV-related liver disease changes and constructed a prognostic gene marker composed of these genes. It is worth noting that these nine genes are all identified as dangerous prognostic genes. Among them, PTTG1, MAD2L1, CDK1, and NEK2 are also the key genes obtained from the protein interaction network. They may be the key risk prognostic genes for hepatitis B inflammation and cancer transformation and play a key role in the progression of hepatitis B to hepatitis B-related HCC. In addition, DEPDC1, ZWINT, PCLAF, RRM2, and TPX2 have also been identified as dangerous prognostic genes. DEPDC1 overexpression promotes HCC cell proliferation, colony formation, and invasion (Guo et al., 2019). Studies have shown that high DEPDC1 expression is an independent predictor of cancer-related death and recurrence. The high expression of DEPDC1 in non-tumor liver is an independent risk factor for late relapse (Amisaki et al., 2019). ZWINT is part of the MIS12 complex, which is necessary for mitochondrial formation and spindle checkpoint activity (Musio et al., 2004). The dysregulation of ZWINT enhanced the chromosomal instability in tumorigenesis and contributed to poor prognosis in malignancies (Pérez de Castro et al., 2007). PCLAF acts as a PCNA-binding protein for DNA repair regulators during DNA replication (Kais et al., 2011). Studies have confirmed that overexpression of PCLAF in adrenal cortical tumors, nasopharyngeal carcinoma, and hepatocellular carcinoma may promote the growth and invasion of cancer cells (Jain et al., 2011; Abdelgawad et al., 2016; Ma et al., 2020). RRM2 is a key protein for DNA synthesis and repair, which can promote cell proliferation and inhibit apoptosis. In previous studies, it has been demonstrated that inhibition of RRM2 significantly inhibits the proliferation of liver cancer cells (Wang et al., 2018). TPX2 is a microtubule-associated protein that involves targeting the kinesin Xklp2 to microtubules. The expression of TPX2 in tumor tissues is higher than that in non-tumor tissues. Overexpression of TPX2 is positively correlated with poor prognosis (Liang et al., 2015).

KEGG pathway enrichment shows that key genes are mainly enriched in oocyte meiosis pathway and cell cycle pathway. Interestingly, in GSE83148, the result of pathway enrichment of differential gene KEGG also included oocyte meiosis pathway and cell cycle pathway; in GSE121248, the result of KEGG included the cell cycle pathway. The pathway enrichment results of 22 overlapping differential genes are also very similar to the pathway enrichment results of the two chips. An epidemiological and virological study of occult hepatitis B infection and hepatocellular carcinoma found that HBV DNA integration affects the liver cell cycle and tumor development, and the promotion of cancer-promoting proteins (such as HBx proteins and mutated surface proteins) produces and continues low-grade hepatic necrotizing inflammation. Inflammation can lead to liver fibrosis and cirrhosis, which is the pathogenic mechanism of occult hepatitis B infection-related hepatocellular carcinoma (Li Y. et al., 2019; Mak et al., 2020). In addition, studies have shown that the HBx gene can be expressed at the one-cell and two-cell stages of embryonic development. The data shows that sperm may be used as a carrier for the vertical transmission of HBV DNA to the next generation (Ali et al., 2005).

## Conclusion

In general, through biological information research methods, we have identified five key genes and nine dangerous prognostic genes. Among them, PTTG1, MAD2L1, CDK1, and NEK2 may be the key prognostic genes of the hepatitis B inflammation and cancer transformation. However, since our research is based on data analysis, further experiments are needed to confirm. At the same time, we hope that our research results have a certain guiding significance for the prognosis and treatment of liver disease in hepatitis B progression.

## Data Availability Statement

Publicly available datasets were analyzed in this study. This data can be found here: https://www.ncbi.nlm.nih.gov/geo/query/acc.cgi?acc=GSE83148 and https://www.ncbi.nlm.nih.gov/geo/query/acc.cgi?acc=GSE121248.

## Author Contributions

JZ and XKL contributed to the conception and design of the study. WZ and SL organized the database. CW performed the statistical analysis. JZ and ZW wrote the first draft of the manuscript. RL, XJL, and JW supervised the project and acquired the funding. YL, SG, and SJ wrote sections of the manuscript. XZ and MW critically reviewed the manuscript for important intellectual content. All the authors contributed to the manuscript revision and read and approved the submitted version.

## Conflict of Interest

The authors declare that the research was conducted in the absence of any commercial or financial relationships that could be construed as a potential conflict of interest.

## Publisher’s Note

All claims expressed in this article are solely those of the authors and do not necessarily represent those of their affiliated organizations, or those of the publisher, the editors and the reviewers. Any product that may be evaluated in this article, or claim that may be made by its manufacturer, is not guaranteed or endorsed by the publisher.

## Footnotes

1
^1^ http://www.ncbi.nlm.nih.gov/geo/

2
^2^ https://cran.r-project.org/doc/FAQ/R-FAQ.html#Citing-R

3
^3^ http://sangerbox.com/TcgaDown

4
^4^ http://sangerbox.com/AllTools?tool_id=9699507

5
^5^ https://david.ncifcrf.gov/

6
^6^ http://sangerbox.com/AllTools?tool_id=9698327

7
^7^ https://string-db.org/

8
^8^ http://www.cytoscape.org/

9
^9^ http://sangerbox.com/AllTools?tool_id=9698305

10
^10^ http://gepia.cancer-pku.cn/index.html

11
^11^ https://icgc.org

12
^12^ https://dcc.icgc.org/projects/LIRI-JP

## Supplementary Material

Supplementary Table 1 The results of the differential analysis of the GSE83148 data set.

Supplementary Table 2 The results of the differential analysis of the GSE121248 data set.

Supplementary Table 3 The clinical characteristics of all patients.

Supplementary Table 4 The results of DEGs analysis of TCGA.

Supplementary Table 5 GO analysis of overlapping DEGs induced by HBV.

Supplementary Table 6 KEGG analysis of overlapping DEGs induced by HBV.

Supplementary Table 7 Prognostic value of the nine genes in the HBV-related HCC patients of the TCGA cohort.

Supplementary Table 8 Sample data of primary solid tissue tumors in ICGC.

## Abbreviations

BP: biological process
CC: cell component
CCNA2: Cyclin-A2
CDK1: Cyclin-dependent kinase 1
DEGs: differentially expressed genes
DEPDC1: DEP domain-containing protein 1A
FDR: false discovery rate
GEO: Gene Expression Omnibus database
GEPIA: Gene Expression Profiling Interactive Analysis
GO: Gene Ontology
HBV: hepatitis B virus
HCC: Hepatocellular Carcinoma
KEGG: Kyoto Encyclopedia of Genes and Genomes
MAD2: Mitotic arrest defect protein 2
MAD2L1: Mitotic spindle assembly checkpoint protein MAD2A
MCODE: Molecular Complex Detection
MF: molecular function
NEK2: Serine/threonine-protein kinase Nek2
NEK2: Serine/threonine-protein kinase Nek2
OS: overall survival
PCLAF: PCNA-associated factor
PPI: protein-protein interaction
PTTG1: Securin
RRM2: Ribonucleoside-diphosphate reductase subunit M2
TCGA: the Cancer Genome Atlas Projec
TPX2: Targeting protein for Xklp2
ZWINT: ZW10 interactor.
